# Multifactorial Model and Treatment Approaches of Refractory Hypotension in a Patient Who Took an ACE Inhibitor the Day of Surgery

**DOI:** 10.1155/2013/723815

**Published:** 2013-04-10

**Authors:** Karan Srivastava, Vikas Y. Sacher, Craig T. Nelson, John I. Lew

**Affiliations:** ^1^Department of Surgery, University of Miami Miller School of Medicine, Miami, FL 33136, USA; ^2^Department of Anesthesiology, University of Miami Miller School of Medicine, Miami, FL 33136, USA; ^3^University of Miami Leonard M. Miller School of Medicine and DeWitt Daughtry Family Department of Surgery, Division of Endocrine Surgery, University of Miami and Jackson Memorial Hospitals, 1120 NW 14th Street, CRB-Room 410P (M-875), Miami, FL 33136, USA

## Abstract

In the field of anesthesiology, there is wide debate on discontinuing angiotensin-converting enzyme inhibitor (ACEI) and angiotensin receptor blocker (ARB) therapy the day of noncardiac surgery. Although there have been many studies attributing perioperative hypotension to same-day ACEI and ARB use, there are many additional variables that play a role in perioperative hypotension. Additionally, restoring blood pressure in these patients presents a unique challenge to anesthesiologists. A case report is presented in which a patient took her ACEI the day of surgery and developed refractory hypotension during surgery. The evidence of ACEI use on the day of surgery and development of hypotension is reviewed, and additional variables that contributed to this hypotensive episode are discussed. Lastly, current challenges in restoring blood pressure are presented, and a basic model on treatment approaches for refractory hypotension in the setting of perioperative ACEI use is proposed.

## 1. Introduction

Approximately 65 million Americans actively receive antihypertensive agents for elevated blood pressure [1]. During surgery, beta-adrenergic blockers and alpha 2 agonists are routinely continued perioperatively because of their role in protecting the myocardium [2–5]. Additionally, calcium channel blockers are used in the perioperative period because of their reduction in myocardial ischemia, infarction, arrhythmias, and overall mortality [6, 7]. Since angiotensin-converting enzyme inhibitor (ACEI) attenuates the adrenergic response to stressful stimuli in cardiac, vascular, and cerebrovascular patients, ACEI is strongly recommended prior to and during these specific surgeries [8–11]. 

**Figure 1:**
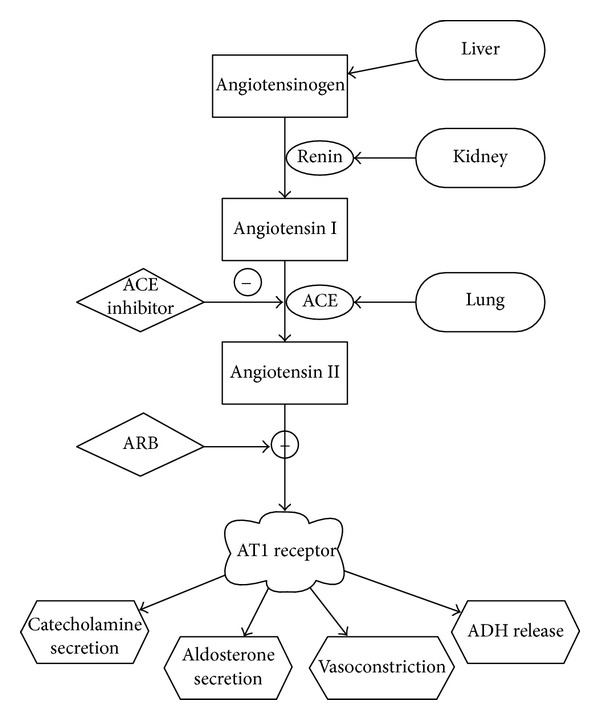
Physiology of the renin-angiotensin system and how angiotensin-converting enzyme inhibitors and angiotensin receptor blockers reduce blood pressure.

However, the use of ACEI and angiotensin receptor blocker (ARB) therapy in the preoperative period in noncardiac patients has been controversial because of its potential role in causing hemodynamic instability. Patients on chronic ACEI or ARB therapy have a dampened sympathetic response [8]. Additionally, surgical patients can be volume depleted because of preoperative fasting, and this condition can cause additional stress during surgery. These combining factors result in reduced vascular capacitance and venous return, leading to decreased cardiac output and subsequent hypotension. To compensate for this hypotension, angiotensin II (ANG2) plays an important role in maintaining blood pressure through vasoconstriction. This vasoconstriction shunts blood away from the kidneys, bowels, and spleen [12, 13]. ANG2's short-term effect is to maintain blood pressure through vasoconstriction whereas its long-term effect, which takes hours to days, is volume regulation through sodium and water retention.  Figure 1 explains the renin-angiotensin system.

Patients who have recently taken ACEI or ARB prior to surgery are unable to use ANG2 effects to counterbalance this hypotension [12]. Compounding this problem is that anesthetic agents have been shown to competitively inhibit ANG2 in rat models [14]. Since there are multiple factors dampening the physiologic response to hypotension in surgical patients who chronically use ACEI therapy, there is a wide discussion as to whether to continue this medication on the day of surgery. 

## 2. Case Report 

A 70-year-old African American female with a left thyroid nodule with an indeterminate FNA result presented for left thyroid lobectomy with isthmusectomy. Her past medical history was significant for hypertension and dyslipidemia. The patient's past surgical history was significant for hysterectomy with no history of anesthesia complications during her prior surgery. Her medication use included lisinopril 40 mg and hydrochlorothiazide 25 mg. She only took lisinopril 40 mg on the day of the surgery. The patient's admission blood pressure was 157/79.

In the premedication stage of general anesthesia, the patient was given midazolam 2 mg. Noninvasive blood pressure, heart rate, and O_2_ saturation were continuously monitored prior to surgery and during surgery. Ten minutes prior to induction, her blood pressure was 150/75. In the induction stage of general anesthesia, the patient was given fentanyl 125 mcg, lidocaine 100 mg, propofol 180 mg, and succinylcholine 100 mg. There were no complications in establishing oral endotracheal intubation. Anesthesia was maintained with sevoflurane. Hypotension (92/54) was first noted 6 minutes after induction. Patient was subsequently given 100 mcg of phenylephrine. She remained hypotensive for the next 120 minutes, despite receiving a total of 1250 mcg of phenylephrine and 90 mg of ephedrine. During her hypotensive episode, the patient's pulse fluctuated from 57 to 95, and she was noted to have a very weak radial pulse bilaterally. At 70 minutes after induction, patient's blood pressure reached its nadir of 63/42 and surgery was halted. When the blood pressure improved slightly with systolic blood pressure in the 70s, surgery was subsequently restarted and completed. The patient's blood pressure was restored to 120/80s in the recovery room. V/Q scan obtained ruled out pulmonary embolism. The patient's lisinopril was withheld postoperative day 1 and her blood pressure was monitored. She spent a day in the surgical ICU and made an uneventful recovery. 

## 3. Discussion

In this case report, the patient continued her ACEI therapy the day of the surgery, while withholding all other medications. Many studies confirm the relationship between hypotension in patients who receive ACEI the same day as surgery. Coriat et al. found that the incidence of induction-induced hypotension necessitating administration of ephedrine was higher in patients who received ACEI the day of surgery compared to patients who had ACEI withdrawn the day prior [15]. Comfere et al. studied the incidence of hypotension in patients who took their last dose of ACEI or ARB less than 10 hours prior to induction, and in patients who took their last dose of ACEI or ARB more than 10 hours prior to induction [16]. Moderate hypotension was defined as systolic blood pressure less than 85 mmHg and severe hypotension as less than 65 mmHg. Patients who received ACEI or ARB less than 10 hours prior to anesthesia had an increased likelihood of developing moderate hypotension only during the first 30 minutes. There was no significant difference in the development of severe hypotension in either group. Rosenman et al. conducted a meta-analysis studying the effect of continuing ACEI and ARB up to the morning of nonemergent surgery [17]. The data selection consisted of 5 studies totaling 434 patients. The meta-analysis found that patients who received the morning dose of ACEI/ARB had a statistically significant increased incidence of perioperative hypotension requiring vasopressors. 

Although ACEI played a role in the development of hypotension in this patient, there are other factors that contributed to her hypotension. Although previous studies have indicated a link between intraoperative hypotension and same-day ACEI therapy, there are other variables confounding these results.Da Costa et al. published a case control retrospective study that found an association between ACEI and hypotension after induction using univariate analysis [18]. However, stratified analysis did not find a statistical significance for an association between ACEI and hypotension. The study showed that when parameters such as age and patient size were controlled for, the use of ACEI was not found to be a dominant risk factor in development of hypotension whereas age was found to be a significant risk factor. 

Other studies have tried to address the role of polypharmacy and the development of hypotension perioperatively. Kheterpal et al. performed a prospective, observational study that showed a synergistic hemodynamic effect in patients who are taking ACEI and diuretic therapy. Patients on chronic ACEI/ARB and diuretic therapy had more episodes of hypotension and required vasopressor boluses more often than the patient solely on chronic diuretic therapy [19]. The study also identified 3 groups of patients on chronic ACEI/ARB therapy who did not have a statistically significant increase in the number of hypotensive episodes. These groups included patients who were on chronic ACEI/ARB but were not taking diuretics or calcium-channel blockers patients who were on chronic ACEI and taking both diuretics and calcium-channel blockers, and patients who were taking chronic ACEI along with calcium-channel blockers. There was no explanation as to why these 3 groups of patients did not have increased incidence of hypotensive episodes. The analysis of polypharmacy interactions of antihypertensive agents is still in the nascent stages. As more pharmacology research is performed and more sophisticated models are developed, physicians answer on whether to continue antihypertensive agents the day of the surgery. 

Anesthetic agents also alter a patient's ability to compensate for hemodynamic instability [20]. Weisenberg et al. did a prospective randomized trial that studied the relationship between the dosage of propofol and the degree of hypotension in patients chronically taking ACEI [21]. Patients were randomly assigned to different propofol dose groups: 1.3, 1.6, 2.0, or 2.3 (mg/kg). A multivariable negative binomial regression model indicated that for each propofol dose increase of 0.3 mg/kg there was an associated 31% increase in mean number of hypotensive/bradycardic episodes. The study also found that the propofol dose of 1.3 mg/kg required the least number of interventions for hypotension or bradycardia during the first 10 minutes after induction. Lastly, the study suggested that adjustments to propofol dose might be effective in reducing perioperative hemodynamic disturbances in patients who chronically take ACEI. 

**Figure 2:**
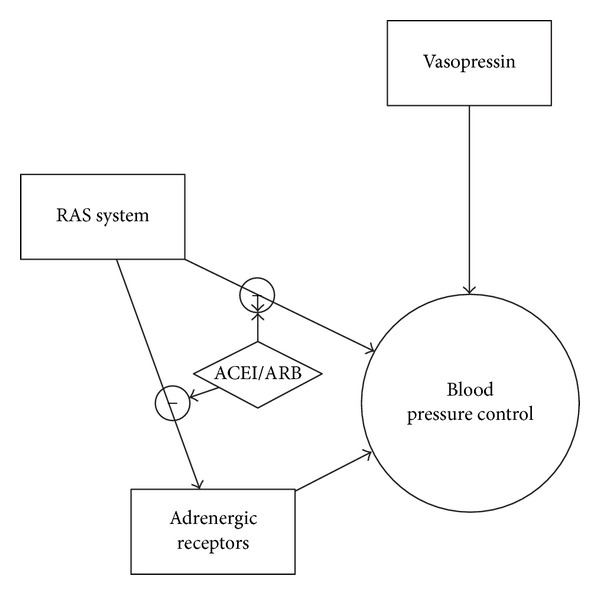
A Description of how ACEI/ARB block the RAS system and adrenergic pathway allowing only vasopressin to regulate blood pressure. (angiotensin-converting enzyme inhibitor) ACEI; (angiotensin receptor blocker) ARB.

**Figure 3:**
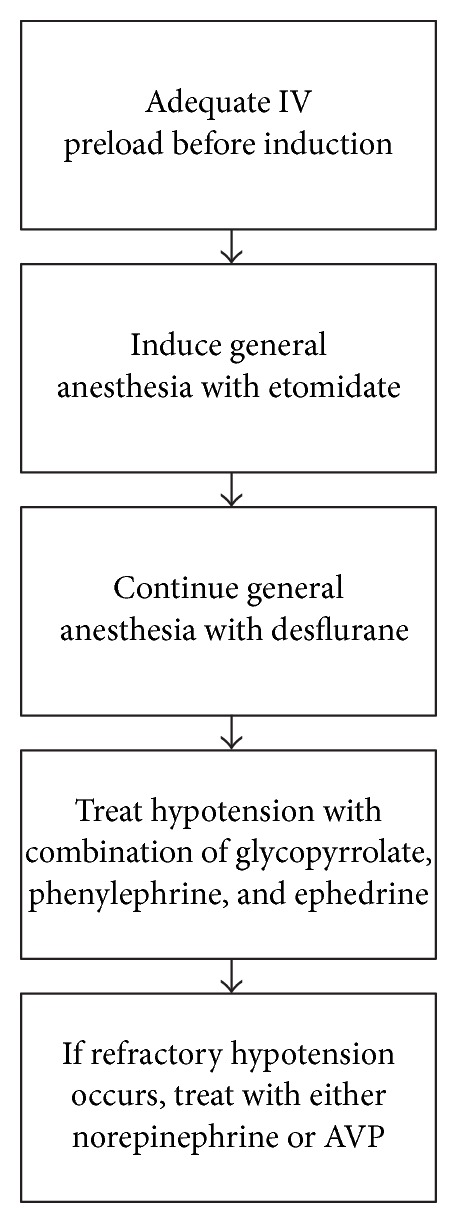
Protocol for refractory hypotension.

Although many variables may have contributed to the patient's hypotension, what is most puzzling about this case is that the patient's hypotension was refractory despite administration of phenylephrine and ephedrine. There is no standard treatment protocol for perioperative hypotension. Nevertheless, the most common treatment approach is the following. First, the depth of anesthesia is reduced and IV fluid is administered. If hypotension persists, phenylephrine, ephedrine, or epinephrine is administered [22]. Wheeler et al. described a case in which a patient on chronic ARB remained hypotensive during surgery despite aggressive administration of phenylephrine, ephedrine, and epinephrine. Eventually, vasopressin was successful in restoring the patient's blood pressure. The authors describe the phenomenon of catecholamine resistance resulting in refractory hypotension in a patient on chronic ACEI therapy. Rat models have further corroborated this possibility. Godínez-Hernández et al. found that ACEI treatment leads to a reduction in alpha-adrenergic receptors density in rats. Even when phenylephrine was administered, there was a 60% decrease in aortic contractility compared to rats not treated with an ACE inhibitor [23]. Since blocking the RAS system disrupts adrenergic receptors, studies have indicated that a possible treatment for refractory hypotension is vasopressin. The vasopressin pathway remains intact even with ACEI or ARB therapy [22, 23].  Figure 2 explains the physiology behind the treatment model for refractory hypotension. The most recent suggested treatment protocol for hypotension in patients on chronic ACEI or ARB therapy is shown in Figure 3 [24]. Thus, there are multiple factors that contribute to the development of refractory hypotension in this patient including age, use of ACEI on the day of surgery, chronic use of ACEI and diuretic therapy as antihypertensives, and the dose of propofol administered at the time of induction. Furthermore, this case suggests and emphasizes that management of refractory hypotension in the setting of ACEI use on the day of surgery should also include the use of vasopressin.

**Figure 4:**
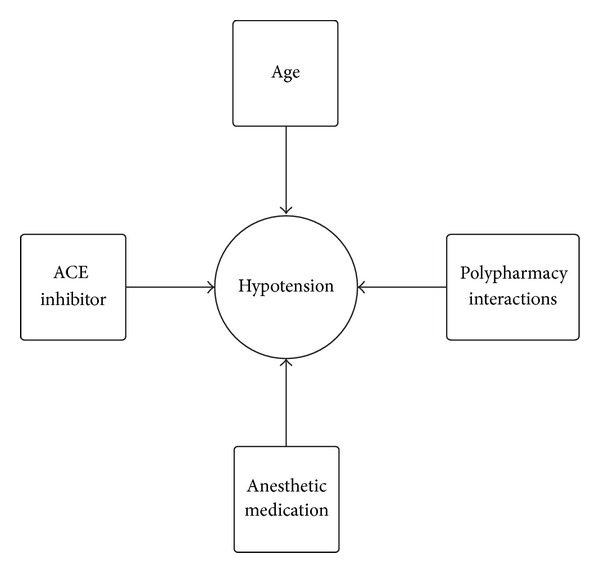
Multiple variables causing hypotension in this case. These models provide a paradigm on how to evaluate patients with perioperative hypotension.

Although this report initially appears to be a simple case of hypotension caused by ACEI treatment on the same day as surgery, there are many additional variables that possibly contributed to hypotension. In this case, the patient was a 71-year-old female on chronic ACEI and diuretic therapy. The patient took her ACEI therapy the day of the surgery. During induction, she was given a total of 180 mg of propofol, which amounts to 2.5 mg/Kg.  Figure 4 is a dynamic model describing the numerous variables that have an effect on blood pressure. These models help illustrate the multifactorial causes of perioperative hypotension. Using such models will be beneficial as more detailed studies describe the precise pharmacology causing perioperative hypotension.
